# Biochemical, molecular and anti-tumor characterization of L-methionine gamma lyase produced by local *Pseudomonas* sp. in Egypt

**DOI:** 10.1016/j.sjbs.2023.103682

**Published:** 2023-05-12

**Authors:** Azza A. Abou Zeid, Asmaa H. Mohamed, Ashraf S.A. El-Sayed, Ashraf M. EL-Shawadfy

**Affiliations:** Botany and Microbiology Deparetment, Faculy of Science, Zagazig University, Zagazig, Egypt

**Keywords:** *Pseudomonas* sp. L-Methionine γ-lyase, Biochemical properties, Molecular characterization antitumor activity

## Abstract

A soil inhabiting *Pseudomonas* sp. has been examined for producing L- methionine gamma-lyase enzyme. The identity of the tested bacteria was verified by VITEK2, and MALDI-TOF analysis in addition to molecular confirmation by 16S rDNA sequence and submitted in Genbank under accession number **ON993898.1.** Production of the targeted enzyme was done using a commercial medium including L-methionine, as the main substrate. This obtained enzyme was precipitated using acetone (1:1v/v) followed by purification with Sephadex G100 and sepharose columns. The specific activity of the purified enzyme (105.8 µmol/ mg/min) increased by 1.89 folds after the purification steps. The peptide fingerprint of the native MGL was verified from the proteomics analysis, with identical conserved active site domains with database-deposited MGLs. The molecular mass of the pure MGL denatured subunit was (>40 kDa) and that of the native enzyme was (>150 kDa) ensuring their homotetrameric identity. The purified enzyme showed absorption spectra at 280 nm and 420 nm for the apo-MGL and PLP coenzyme, respectively. Amino acids suicide analogues analysis by DTNB, hydroxylamine, iodoacetate, MBTH, mercaptoethanol and guanidine thiocyanate reduced the relative activity of purified MGL. From the kinetic properties, the catalytic effectiveness (*K_cat_/k_m_*) of *Pseudomonas* sp*.* MGL was 10.8 mM ^-1^ S^-1^ for methionine and 5.51 mM ^-1^ S^-1^ for cysteine, respectively. The purified MGL showed highly significant antiproliferative activity towards the liver carcinoma cell line (HEPG-2) and breast carcinoma cell line (MCF-7) with half inhibitory concentration values (IC_50_) 7.23 U/ml and 21.14 U/ml, respectively. No obvious signs of toxicity on liver and kidney functions in the examined animal models were observed.

## 1 Introduction

The enzyme Methioninase (MGL, EC 4.4.1.11), which is dependent on pyridoxal 5′-phosphate, catalyzes the transformation of L-methionine into ketobutyrate, methanethiol and ammonia (Tanaka et al., 1977). L-Methioninase has drawn a lot of interest for its essential anti-proliferative effect against several types of cancer cell lines **(**Cellarier et al., 2003, Bopaiah et al., 2020**)**. The lack of methionine synthase activity in tumors, a distinct metabolic criterion that explains the reliance of cancer cells on extrinsic plasma L-methionine, elaborates the rationality of MGL's focused anticancer effect (Sato et al., 2008, Kudou et al., 2007**)**. Unlike normal cells, methionine dependency is a physiological criterion for various tumor cells including prostate, melanoma, colon, kidney and fibrosarcoma cells (Tanaka et al., 1977, Goyer et al., 2007, Hoffman, 2015**)**. Thus, methionine depletion selectively represses the different biological processes of tumor cells particularly cellular gene methylation, cell cycle transition, and cellular protein synthesis (Martinez-Cuesta et al., 2006**)**, preventing cells from dividing at the G2 phase and resulting in cell death **(**Tanaka et al., 1977, Sato et al., 2006, Goyer et al., 2007, El-Sayed et al., 2017**)**.

Methionine γ-lyase has been reported to be produced by various fungal and bacterial isolates. The enzyme was purified from a number of bacterial isolates including *Aeromonas* sp., *Pseudomonas putida*; *Brevibacterium linens*; *Citrobacter freundii*; *Lactococcus lactis* as well as *Clostridium sporogenes* (Tanaka et al., 1985, Tanaka et al., 1977, Amarita et al., 2004, Manukhov et al., 2005, Martínez-Cuesta et al., 2006, Morozova et al., 2017**)**. As well as, MGL has been extensively characterized by *Aspergillus flavipes* (Khalaf et al., 2009). Comparative biochemical and spectroscopic studies of MGL from *P. putida* and *A. flavipes* were reported **(El Sayed *et al.,* 2017)**. From the crystal analyses, MGL from *C. freundii, P. putida* (**El Sayed, 2010** and El-Sayed et al., 2017**)** and *A. flavipes* **(**El-Sayed et al., 2017) has a PLP coenzyme via the internal aldimine linkage with the enzyme's lysine residues' ε-amino group. The promising therapeutic activity of bacterial MGL had been reported by Kudou et al. (2007). Several trials have been proposed to enhance the stability of the structure and reduce the antigenicity of MGL from different microbial sources **(** Vachher et al., 2021, El-Sayed et al., 2017**)**. Purified MGL from *A. flavipes* was chemically modified by polyethylene glycol, dextran and co-immobilized with glutaraldehyde dehydrogenase to scavenge the toxicity of released ammonia (El-Sayed et al., 2014). However, catalytic effectiveness and conformational stability are still the major challenges for further clinical trials of MGL. Thus, the characterization of pure MGL with unique biochemical properties especially higher catalytic efficiency and structural stability from novel microbial isolates was the goal of this research.

## 2 Materials and methods

### 2.1 Production of MGL enzyme by tested bacteria

A Gram-negative bacteria collected from a non-cultivated soil sample in Daqahlia Governorate, Egypt, was isolated and purified on nutrient agar media. The potency of isolated bacteria for producing MGL was evaluated by the modified method of **Esaki and Soda (1987)**. Briefly, the medium includes 0.25 % L-methionine, 0.1 % polypeptone, 0.1 % glycerol, 0.1 % KH_2_PO_4_, 0.1 % K2HPO4, 0.01 % MgSO_4_·7H_2_O, and 0.02 % yeast extract. A loop full of the bacterial isolate was inoculated to 100 ml Erlenmeyer conical flask with 25 ml of medium, then kept at 37 °C for three days at 120 g. Bacterial pellet was collected by centrifugation for 10 min at 5000 g, then washed with 10 mM sterile phosphate buffer (pH 7.8).

The cellular crude protein was extracted according to Manukhov et al. (2006). The bacterial cells (0.5 g) were suspended in 5 ml (10 mM) phosphate buffer (pH 7.8) with 0.5 mM phenylmethylsulfonyl fluoride (PMSF) and pulverized for a four-minute by ultrasound under cooling. The homogenate was aggressively stirred for about 15 min, then centrifuged at 10,000g for 10 min under cooling at 4 °C to get rid of cell debris. The obtained supernatant served as a crude source of crude intracellular MGL, and the activity of enzyme and its concentration were measured.

### 2.2 Activity and concentration of MGL

#### 2.2.1 Demethiolating activity of MGL

The demethiolating activity of MGL was calculated from the amount of liberated methanethiol by using the 5,5-dithiol-bis-2-nitrobenzoic acid (DTNB) assay (Laakso and Nurmikko, 1976**)**. Briefly, the components of the reaction include phosphate buffer with 1 ml of 20 mM L-methionine (pH 7), 10 mM PLP, and 0.25 mM DTNB reagent. Separate blanks for the substrate and the enzyme were prepared. The reaction was kept warm for 30 min at 37 °C, and the released yellow coloring was assessed at λ420 nm. The quantity of resulting methanethiol served as a proxy for the activity of MGL, which was calculated using DTNB under identical circumstances using a standard curve with varying cysteine concentrations. Under ideal test circumstances, the quantity of enzyme that introduces 1 M of methanethiol/minute was expressed by one unit (U) of MGL. The enzyme activity (U) per mg protein served as a measure of the enzyme's specific activity.

#### 2.2.2 Deaminase activity of MGL

The direct Nesslerization test was used to quantify the MGL activity. The assay combination in a total volume of 0.5 ml, l-methionine (10 mM) was added, PLP(0.02 mM), phosphate buffer (50 mM)(pH 7.8), and>0.015 U of the enzyme **(**Nakayama et al., 1984**).** After 1 h of incubation at 25 °C, the mixture was halted with 5% Tri carboxylic acid (TCA). The mixture was whirled for ten minutes at 5000 g, and then a volume of 50 µl of Nessler's reagent was added. This evolved coloration was determined at about 500 nm after 15 min. Under ideal test circumstances, the quantity of enzyme required to produce one mole of ammonia/minute was used to represent one unit of MGL.

#### 2.2.3 Concentration of enzyme

Folin's test (Lowry et al., 1951) was used to assess the quantity of enzyme protein using bovine serum albumin as the reference.

### 2.3 Biochemical and molecular identification of tested MGL-producing bacterial isolate

The bacterial isolate was identified based on its biochemical parameters (Garrity, 2005**)**. The bacterial isolate was identified using the VITEK automated system (Ling et al, 2003**)** located at 57,357 Children’s Cancer Hospital Foundation, Egypt and the MALDI-TOF MS spectrometer, Autof MS2000 system **(**Singhal et al., 2015**)**, located at 57,357 Children’s Cancer Hospital Foundation, Egypt. Identity of tested bacteria was confirmed using 16S rDNA sequence using the universal primer 5′-27F (5′AGAGTTTGATCCTGGCTCAG3′) and 1492R(5′GGTTACCTTGTTACGACTT3′) **(**de Lillo et al., 2006**)**. Re-suspended bacterial colonies in 50 ml of lysis solution, which contains Tris-HCl (10 mM), Tween 20 (0.1%), and KCl (50 mM) (pH 8.3), were incubated for 10 min at 99 °C. The bacterial lysate (1 μl aliquot) was utilized as a PCR template for amplification. The PCR reaction is composed of 10 µl of the master mixture (2x) (i-TaqTM, Cat. No. 25027, iNTRON Biotech.), 1 µl of forward and reverse primers (10 pmol), 2 µl of bacterial lysate and Polymerase chain reaction committed to 20 µl total volume with sterile distilled water. The PCR was set up to have 35 cycles, starting with denaturation at 94 °C for 2 min, denaturation at 94 °C for 30 s, followed by annealing at 55 °C for 10 s, extension at 72 °C for 30 s, with a final extension at 72 °C for 2 min.

The PCR amplicons were assessed in comparison to a DNA ladder (1-kilo base pair Nex-gene Ladder, Puregene, Cat.# PG010-55DI) using agarose gel (1.5%) in Tris/Borate/EDTA(TBE) buffer (Ambion Cat# AM9864).

Negative controls devoid of bacterial genomic DNA were employed. A gel documentation system was used to analyze the amplicons. Using the same primer sets, Applied Biosystems Sequencer, HiSQV Bases, Version 6.0. was used to sequence the amplicons after purifying them. The obtained 16 s rDNA sequence was BLAST-searched on the NCBI database without redundancy. The sequence chromatograms were used to visually examine the quality of the retrieved sequences. FASTA sequences were input into MEGA 6.0 software for the multiple sequence alignment, and the ClustalW muscle algorithm was used to produce phylogenetic tree (Edgar and Muscle, 2004**).** Target sequences' of the phylogenetic tree was produced by MEGA 6.0′s neighbor-joining approach with 1000 bootstrap replications (Tamura et al., 2011).

### 2.4 MGL purification, subunit structure of MGL and molecular mass from tested bacteria

The robust bacterial isolate that produces MGL was cultivated in a commercial medium containing methionine **(**Esaki and Soda 1987**)**. The bacterial pellets (20 g) were collected after incubation and washed with sterile distilled water and the intracellular crude protein was extracted according to Manukhov et al. (2006). The crude enzyme's activity and protein content in the collected supernatant was assessed as mentioned before. The acetone precipitation method was used to purify the enzyme **(**Nejadi et al., 2014**)**. Five volumes of pre-chilled absolute acetone (at −20 °C) were added to one volume of the sample and kept for 15–20 min then vortexed for 30 s. The precipitate was separated by centrifugation at 10,000 g for 10 min. Protein pellets were collected, and 10 ml potassium phosphate buffer (pH 7.5, 50 mM) was used to dissolve them. By using dialysis bag with a 20 kDa cut-off (Cat. # 546–00051, Wako Chemicals, USA), the precipitated proteins were selectively concentrated against polyethylene glycol 6000 till reached 2 ml total volume. By using gel filtration and ion exchange chromatography, the enzyme was purified (Tanaka et al., 1976, Selim et al., 2015**)**. A sephadex column G200 (240 cm) was loaded by the crude protein after being pre-equilibrated with 50 mM potassium phosphate buffer (pH 7.5) at a rate of flow 0.5 ml/min. The same buffer was used to elute the enzyme fractions (1 ml), and the standard test was used to assess the enzyme activity and concentrations. Dialysis against polyethylene glycol 6000 was used to collect and concentrate the most active fractions. The first step partially purified enzyme was further refined using ion exchange chromatography column of DEAE-sepharose (2 × 30 cm), with potassium phosphate buffer pre-equilibrated (0.1 mM, pH 7.5). Following gel permeation, the column was loaded with the sample and then equilibrated with a flow rate of 1 ml/min through the same buffer. This buffer containing gradient concentrations of NaCl (100–300 mM) was used to elute the enzyme. The recovered fractions' activity and protein content were assessed as previously mentioned. The MGL fractions with the highest activity were gathered and concentrated using a 10 K ultra-centrifugal membrane. The pure MGL was held at 4 °C pending assay. The purified MGL's subunit structure and molecular homogeneity were checked by running over 12% SDS-PAGE **(**Laemmli, 1970). Using the same methodology, without SDS in buffers, native-PAGE was carried out.

### 2.5 The bacteria's MGL peptide fingerprinting

The peptide fingerprint of the pure enzyme was examined by the Liquid Chromatography-Tandem Nanospray Ionization Mass Spectrometry (LC-MS/MS) at the Proteomics and Metabolomics Facility Core, 57,357 Children’s Cancer Hospital Foundation, Egypt. The MGL was removed from the SDS-PAGE gel, and the gel fragment was ground before being destained by 200 ml of 50 mM ammonium bicarbonate (AB)-acetonitrile (1:1 v/v) and 500 ml of acetonitrile. After the extra acetonitrile was vacuum-extracted, the gel was re-swollen in 100 mM AB with 10 mM dithiothreitol (DTT) for 30 min at 60 °C, then in 100 mM AB with 50 mM iodoacetamide. Using trypsin, the dried gel fragments were broken down (20 μl Trypsin, porcine MS grade, 10 ng/ µl) for 12 h at 37 °C, under shaking conditions. The supernatant was combined, added to 100 ml of extraction buffer which was made of 5% formic acid and 1:2 acetonitrile, and then kept standing for 15 min at 37 °C. The peptides were desalted and analyzed by the nanospray ionization using Triple-TOF 5600 mass spectrometer, interfaced by nano-scale RP-HPLC (Stadler et al., 2013**)**. Peptide elution to mass spectrometry from a column was carried out using a linear cascade of acetonitrile (ACN) buffer (5–60%) flowing at a rate of 20 L/min. The MS/MS data were obtained independently, and the MS/MS data is being obtained from *m*/*z* for 50–2000 Dalton and MS1 data being obtained for 250 ms at *m*/*z* for 400–1250 Dalton (Stadler et al., 2010, Stadler et al., 2013**)**. After extracting the MS/MS data files, Protein Pilot 4.0 was used to analyze and identify the peptides (ABSCIEX) **(**Inoue et al., 1995**)** matching the *Pseudomonas* sp. proteome. Five peptide segment ions with at least**/**protein of E-values under 0.05 were required for identification.

### 2.6 Biochemical properties of MGL from tested bacteria

#### 2.6.1 Reaction temperature, thermal stability, pH stability and MGL inhibitors

The biochemical characteristics of the purified MGL from the bacteria, including optimum temperature, thermal stability, reaction pH, pH stability and MGL inhibitors were examined. The enzyme reaction mixture was incubated at various degrees of temperatures (30, 37, 40, 45 and 50 °C), and the MGL activity was then detected using the standard test to determine the temperature at which the purified MGL was most active. MGL thermal stability was assessed by keeping at 37, 45 and 50 °C for 30, 60, 120 and 180 min, respectively, and the remaining demethiolating activity of MGL was then assessed by the standard test. Thermal kinetic parameters such as the thermal inactivation rate (*Kr*) and half-life time (*T1/2*) were calculated according to Salim et al. (2020). MGL pH stability was measured using different pH ranges (pH 3.0–5.0 using 0.1 M phosphate-citrate buffer, pH 5.6–7.8 using potassium phosphate buffer and pH 8.0–10.0 using phosphate buffer) for 2 h at 4 °C and then the remaining MGL activity was calculated as previously mentioned. The precipitation's pH was determined by keeping the purified MGL at different pH range (3.0–10.0) for 12 h at 4 °C, then the mixture was centrifuged at 100,000 g for 10 min, and the amount of protein that precipitated was examined by Folin’s reagent (Lowry et al, 1951**)**. The pH where the protein was precipitated most readily, served as an indicator of the isoelectric focusing (*pI*).

To evaluate the metalloproteineous identity of MGL, the enzyme was concentrated using dialysis bag size 20 KDa (Cat# 546–00051, Wako Chemicals, USA) against a 50 mM Tris-HCl buffer (pH 8.0) containing 10 mM EDTA, then enzyme preparations were desalted. Several cations such as K^+^, Na^+^, Zn^2+^, Mg^2+^, Ba^2+^, Ca^2+^, Hg^2+^, Cu^2+^, Al^3+^and Fe^3+^ were introduced to pure MGL at a total concentration of 1 mM (El-Sayed and Shindia, 2011, El-Sayed et al., 2017). Several suicide amino acid analogues (hydroxylamine, DTNB, iodoacetate, MBTH, guanidine thiocyanate and 2-mercaptoethanol) were added to pure enzyme with a total concentration of 1 mM, kept for 2 h at 4 °C, thereafter the remaining enzymatic action was determined using the standard test.

#### 2.6.2 The specificity of the pure MGL towards different substrates

The affinity of MGL for catalyzing the elimination and deamination reactions of various amino acids was evaluated towards L-cysteine, D-glycine, DL-homocysteine, L-asparagine, L-tryptophan, L-ornithine, L-phenylalanine, L-tyrosine and L-arginine employing L-methionine as the standard substrate. MGL activity was measured by keeping the enzyme with 20 mM of the different substrates in phosphate buffer (pH 7.8) of 10 µM PLP, in one ml total volume, and then the demethiolating and deaminating enzyme activity was assayed, as mentioned previously. The catalytic and kinetic parameters such as maximum velocity (*V_max_*), Michalis-Menten constant (*K_m_*) and turnover number (*K_cat_*) were calculated (El-Sayed et al., 2017).

#### 2.6.3 UV spectroscopic analysis of the purified MGL

The intrinsic UV-spectra of the pure enzyme was assessed (Yadav et al., 2012). The purified MGL was added to 100 mM phosphate buffer (pH 7.8) containing 1 mM EDTA. The phosphate buffer (pH 7.8) served as the standard. Meanwhile, the buffer (pH of 7.8) with 1 mM EDTA was used to dissolve the enzyme and served as a baseline for further analyses. By using UV-spectral scanning, the MGL's internal Schiff base was evaluated at the 200–600 nm range (El-Sayed and Shindia, 2011, El-Sayed et al., 2012**)**. Triplicates were conducted for each enzyme preparation.

#### 2.6.4 Antiproliferative effect of bacterial MGL againt tumer cell lines

The antiproliferative activity of the purified l-methioninase was examined against breast carcinoma (MCF7) and Hepatocellular carcinoma (HPG2) at Al-Azhar University, The Regional Center for Mycology and Biotechnology **(**Cory et al., 1991, Mosmann, 1983**)**.

#### 2.6.5 In-vivo toxicity of MGL

Cytotoxic properties of *Pseudomonas* sp. MGL were evaluated in vivo using female Swiss Albino mice weighing 25 g. The experimental protocols were approved by the IAEC (Institutional animal ethical committee – Zagazig University) (approval number: ZU-IACUC/1/F/220/2022) and under the recommendations for the proper care and use of laboratory animals. The mice were intravenously injected using a single dosage (50 µl) of the MGL (50 mg/kg) and allowed to adapt for three days. Three mice are employed in each treatment group. Untreated mice were kept as control group. The biochemical characteristics of blood samples taken via tail vein were analyzed. MGL activity in recovered sera from tested mice was determined using previously mentioned standard test.

#### 2.6.6 Mice blood biochemistry in response to injection with MGL

The collected blood samples from treated and untreated mice were used for the estimation of biochemical parameters, denoting liver and kidney functions, such as AST, APL, ALT, albumin, total protein, globulin, creatinine and urea, as markers to evaluate the toxicity of MGL. These markers were analyzed as follows:

### 2.7 Assay of transaminases

Transaminases such as aspartate aminotransferase (AST) and alanine aminotransferase (ALT) were assessed using SPINREACT Clinical Systems Kit. Briefly, the reaction mixture contained 500 µl working solution (800 U MDH, 0.2 mM NADH, 4000 U LDH, 13 mM α-ketoglutarate and 264 mM L-aspartate added to 88 mM of Tris-HCl (pH 7.8)) for AST assay. For the ALT assay, the working solution was (0.2 mM NADH 16 mM, α-ketoglutarate,1200 U LDH and 550 mM L-alanine added to Tris-HCl (pH 7.5) of 110 mM, then added to 50 µl of serum for each enzyme treatment, comparing to control. The alteration in absorption spectrum was measured per minute at A_340_ nm. AST and ALT activities were calculated according to the instructions of the working kit **(**Berth and Delanghe, 2004**)**.

Activity (μkat/l) = A/min. × 1746 × 0.0167.



#### 2.7.1 Alkaline phosphatase (ALP)

The activity of ALP was determined using SPINREACT Kit. The reaction mixture contained 10 mM P-Nitrophenyl phosphate, 1 mM diethanolamine buffer (pH 9.8), 0.6 mM magnesium ions and 20 µl serum. After one min of incubation, the resulting color was evaluated at A_405_ nm (Young, 1990). The reaction consequenced as follows;



#### 2.7.2 Albumin (ALB)

Albumin was assessed using SPINREACT Kit. The reaction contained 2 ml of working solution (75.0 mM succinate buffer, pH 4.2 with 0.26 mM Bromcresol green) and 10 µl serums and leave for 5 min at room temperature. The developed color was assessed at A_580_ nm (Young, 1990). Serum albumin concentration = A sample/A standard × standard value.



#### 2.7.3 Total protein

Total protein was assessed using SPECTRUM Kit. The reaction mixture contained 1 ml of working solution (750 mM sodium hydroxide, 12 mM copper sulphate, 40.9 mM sodium potassium tartrate and 19.8 mM potassium iodide) and 20 µl of serum. The reaction was kept for 10 min and the released color was detected at A_546_ nm **(**Burtis and Ashwood, 1999**)**. The concentration of protein (gm/dl) = A sample/A standard × 6.



#### 2.7.4 Urea

Urea was assessed using DIAMOND Kit. The reaction included 1 ml of reagent A (phosphate buffer 50 mM (pH 6.7), 2 mM EDTA, 400 mM sodium salicylate and 10 mM sodium nitroprusside), 50 µl of 3000 U Urease and 10 µl of serum and leave at 37 °C for 3 min. Then, 200 µl of reagent B (140 mM sodium hypochlorite and 150 mM sodium hydroxide) was added and kept for 5 min at 37 °C. The resulting coloring was assessed at A_578_ nm (Young, 1990).

Urea concentration = Asample/Astandard × 50 (Standard conc.).



#### 2.7.5 Creatinine

Creatinine was assessed according to the instruction of the SPECTRUM kit. One ml of 25 mM picric acid with 1 ml of 0.4 mM sodium hydroxide was mixed with 100 µl of mice serum and the change in absorbance was measured per min at A_492_ nm **(**Tietz, 1986**)**. The concentration of creatinine (mg/dl) = Asample/Astandard × 2.



#### 2.7.6 Statistics

All data were represented by three replicates and expressed as mean ± STDV. (http://WWW. Easy calculation.com/statistics/fiishers-Isd-calculator).

To confirm the significant differences in all the parameters, the data were statistically analyzed using one-way analysis of variance (ANOVA) using SPSS software (version 20.0) **(**Levesque, 2007**)**.

## 3 Results

The Gram-negative bacterial isolate confirmed as a potent MGL-producing isolate was identified using the automated VITEK- 2 system and analyzing its peptide sequence using the MALDI TOF system, as *Pseudomonas* sp.

**Fig. 1:**
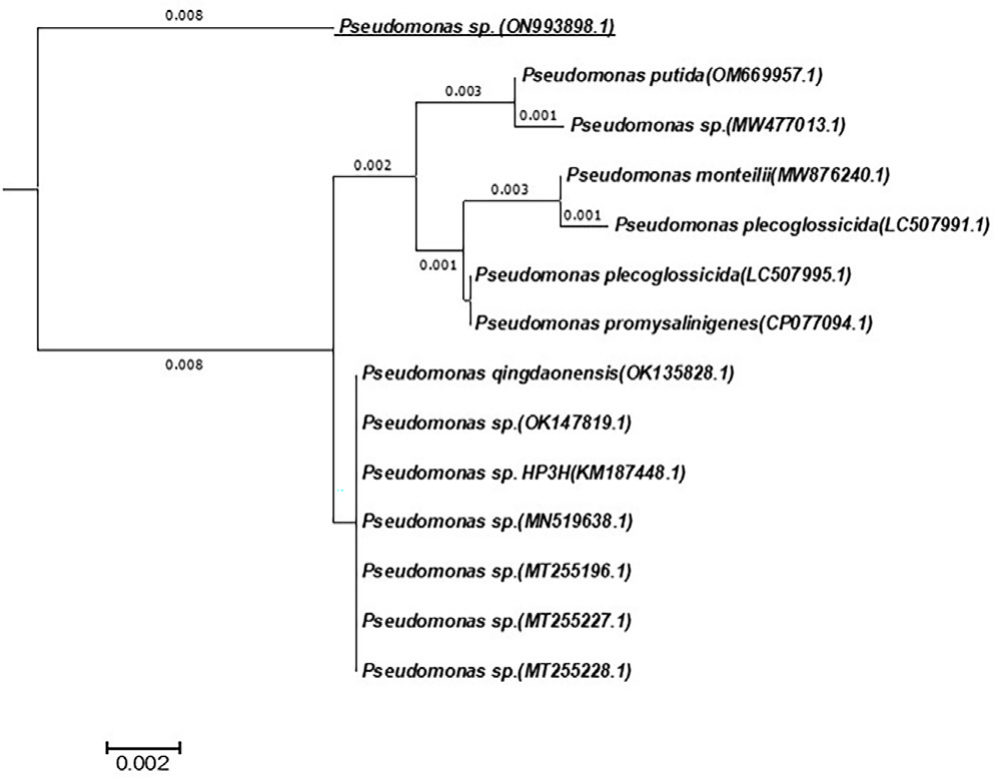
Phylogenetic tree built from the alignment of partial sequence of the 16S rRNA gene of tested isolate showing similarities with 13 sequences confirming its identity as *Pseudomonas* sp. Deposited under accession number ON993898.1.

The identification of the tested isolate was molecularly verified depending on the sequence of the 16S rRNA gene, using its genomic DNA as a template for PCR. The amplicons of PCR were visualized with an apparent size of 1500 bp. The specific PCR amplicon amplified using universal primers was purified, sequenced and non-redundantly BLAST searched on the database. The phylogenetic tree built from the alignment analysis **(**Fig. 1**)** of the obtained sequence revealed that the tested isolate displayed 99 % similarity with *Pseudomonas* species encoded: OM669957.1, MW477013.1, MW876240.1, LC507991.1, LC507995.1, CP077094.1, OK135828.1, 0 K147819.1, KM187448.1, MN519638.1, MT255196.1, MT255227.1, MT255228.1 with E-value zero and query coverage 98%. The result is coincident with the biochemical and metabolic identifications from VITEK 2 and MALDI-TOF identification systems and the isolate is identified as *Pseudomonas* sp. and submitted under accession number (ON993898.1) in Genbank.

### 3.1 Purification and molecular subunit structure of MGL from *Pseudomonas* sp*.*

Methionine gamma-lyase (MGL) enzyme produced by tested *Pseudomonas* sp. after being grown in a commercial nutrient medium described by Esaki and Soda (1987) was further extracted and purified from the harvested bacterial pellets. Total crude intracellular protein from the bacterial isolates was concentrated by acetone precipitation, then dialysis using 20 kiloDalton cut-off membrane, against polyethylene glycol. The purification profile of MGL is presented in Table 1.

**Table 1:** Overall purification profile of MGL from *Pseudomonas* sp*.*

**Step**	**Total Protein (mg/ml)**	***Pseudomonas* sp.**			
		**Total Activity (µmol/min)**	**Specific activity (µmol /mg/min)**	**Purification Fold**	**Yield**
**Crude**	0.085	4.791	56.23	1	100
**Acetone**	0.056	3.24	57.98	1.031	67.77
**Sephadex G100**	0.02	1.93	96.97	1.725	40.48
**DEAE-Sepharose**	0.017	1.8	105.8	1.89	37.5

Obtained results revealed an increase in MGL enzyme activity by 1.725 folds, and a yield of 40.48% after gel-filtration using Sephadex G100. Most active fractions (6–20) were gathered and further eluted by 150 mM NaCl through ion exchange DEAE-sepharose. MGL assessment indicated an increase in enzyme activity by 1.89 folds with a recovery yield of 37.5% with an overall specific activity of 105.8 µmol /mg/min.

### 3.2 Molecular homogeneity, spectroscopic analyses and peptide fingerprint of MGL from P*seudomonas* sp.

The purified MGL enzyme was further checked for its molecular mass and subunit structure by the denatured and native PAGE. The molecular mass of the subunit structure of MGL of *Pseudomonas* sp*.* was > 40 kDa when detected by SDS-PAGE. Meanwhile, the entire molecular mass was ∼ 140–150 kDa, as revealed from the native PAGE. These results emphasize that MGL produced by *Pseudomonas* sp. has four identical subunits in molecular mass and charges (i.e. homotetrameric identity).

Spectroscopic analyses of purified MGL enzyme produced by *Pseudomonas* sp. were determined at wavelengths 200–600 nm. Results in Table 2 revealed maximum absorption of UV-light at wavelength 280 nm followed by wavelength 420 nm recording a high A280/420 ratio of 3:2.

**Table 2:** UV-Spectroscopy of purified MGL enzyme of *Pseudomonas* sp.

**UV (nm)**	**Absorbance (OD)**	**UV (nm)**	**Absorbance (OD)**
**180**	**0**	**320**	**0.05**
**190**	**0**	**340**	**0.04**
**200**	**0.02**	**360**	**0.2**
**210**	**0.03**	**380**	**0.4**
**22**	**0.04**	**400**	**0.59**
**230**	**0.06**	**420**	**1.1**
**240**	**0.2**	**440**	**0.7**
**250**	**1**	**460**	**0.6**
**260**	**1.5**	**480**	**0.4**
**270**	**2.4**	**500**	**0.2**
**280**	**3.5**	**520**	**0.1**
**290**	**1.2**	**540**	**0.1**
**300**	**1**		

**Fig. 2:**
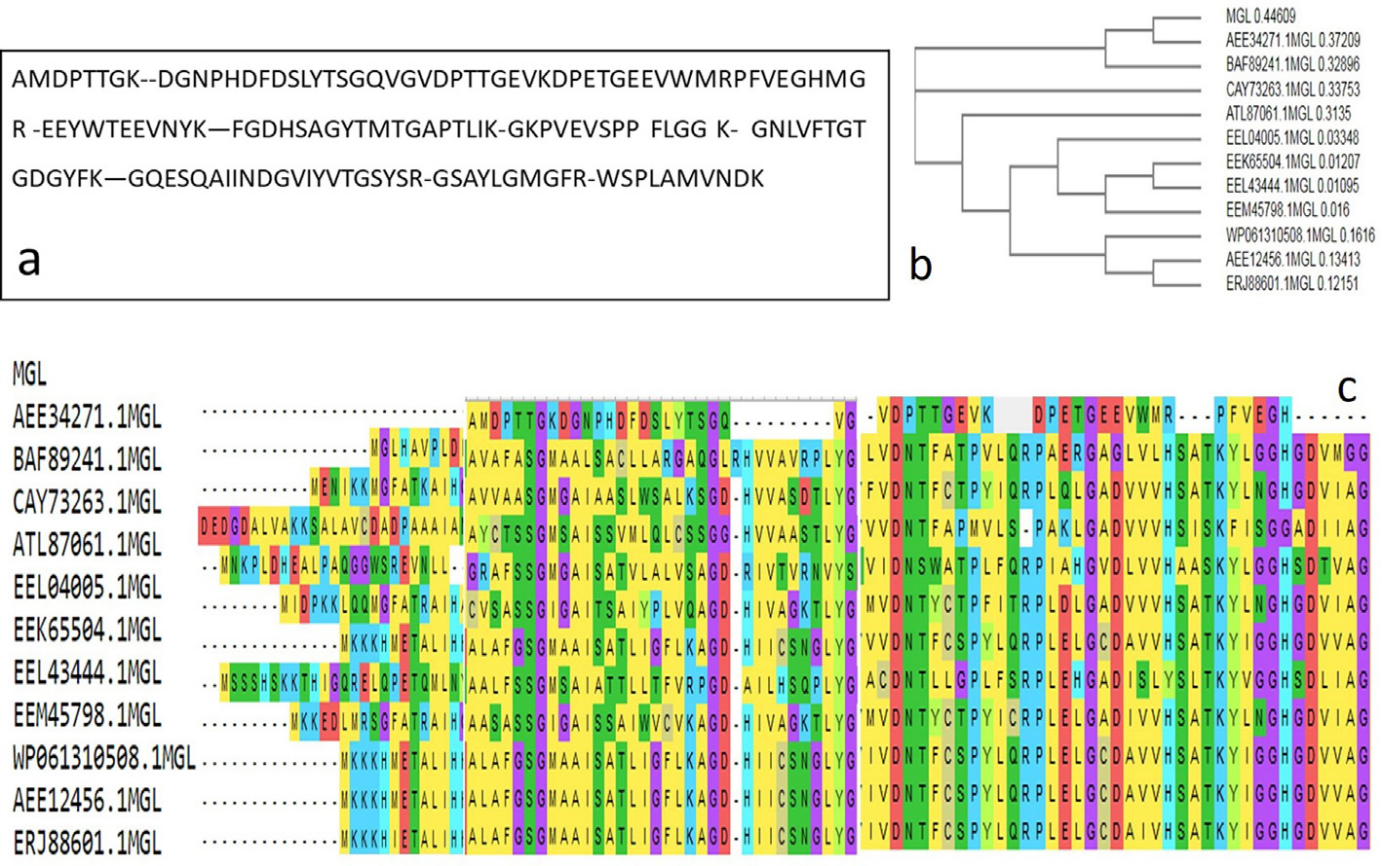
Proteomic analysis of the purified MGL from *Pseudomonas* sp. a) Partial peptide sequence of the MGL from the MALDI-TOF; b) Phylogenetic analysis of amino acids sequence with database deposited MGL sequences; c) Multiple alignment analysis of the peptide fragments of the *Pseudomonas* sp. MGL compared to 11 organisms showing the conservative active sites.

The peptide fingerprint of the purified *Pseudomonas* sp. MGL was assessed by LC-MS/MS, after trypsinization followed by nanospray ionization. The extracted raw peptides MS data were examined, identified, and normalized to the *Pseudomonas* sp. proteome by Protein Pilot 4.0 (ABSCIEX). The identifying criterion required at least five peptide segment ions of E-values < 0.05) per protein. The obtained sequences of peptide were categorized by matching with MGL sequences from various microbial origins (Fig. 2**a&b**). The numerous aligning analysis revealed that the partial peptide sequence of *Pseudomonas* sp*.* MGL had 80% similarity with the MGL from *Streptomyces malaysiensis* ATL87061.1, *C. botulinum* WP061310508.1, *Arabidopsis thaliana* AEE34271.1, *Azorhizobium caulinodans* BAF89241.1, *Porphyromonas asaccharolytica* AEE12456.1, *B. wiedmannii* EEK65504.1, *Erwinia pyrifoliae* CAY73263.1, *Porphyromonas gingivalis* ERJ88601.1, *B. thuringiensis* EEM45798.1, *B. cereus* EEL43444.1 and EEL04005.1.

The phylogenetic evaluation of partial *Pseudomonas* sp. peptide sequence in the database submitted MGLs sequences was observed. Phylogenetic analysis indicated the peptide sequence of tested *Pseudomonas* sp. MGL displays high proximity to *Arabidopsis thaliana* AEE34271.1 and *Azorhizobium caulinodans* BAF89241.1 **(**Fig. 2**b).**

The partial peptide sequence's conserved active sites domains of *Pseudomonas* sp. MGL (Glycine- Aspartic acid-Histidine –Valine) matched with that in MGL from species of *P. asaccharolytica* AEE12456.1, *B. wiedmannii* EEK65504.1, *E. pyrifoliae* CAY73263.1, *P. gingivalis* ERJ88601.1, *B. thuringiensis* EEM45798.1, *B. cereus* EEL43444.1 and EEL04005.1 (Fig. 2**c**).

### 3.3 Biochemical properties of the purified MGL from *Pseudomonas* sp.

#### 3.3.1 The reaction temperature and thermal stability of MGL

**Fig. 3:**
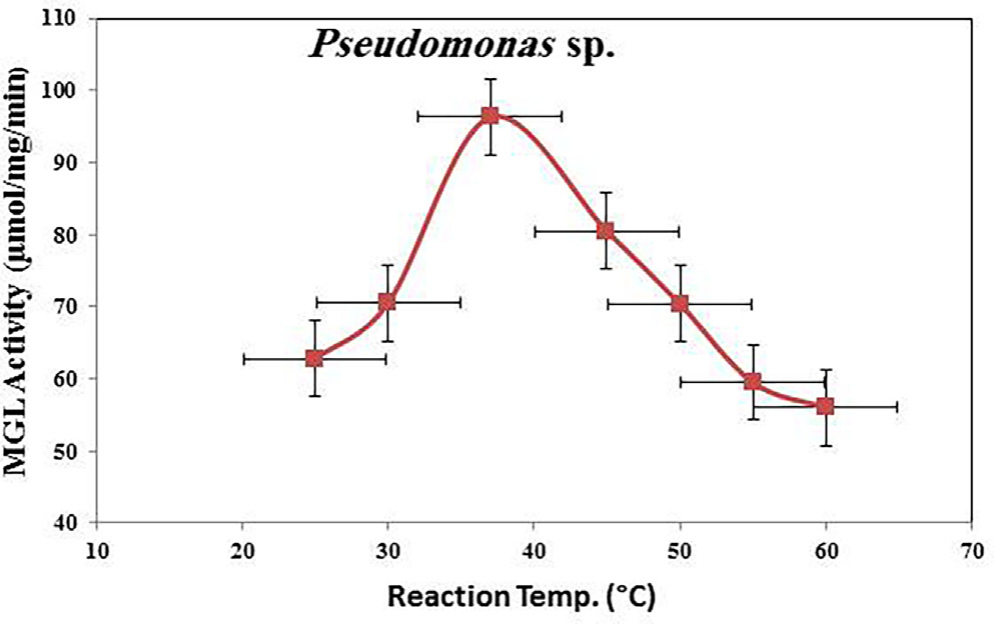
Effect of increasing reaction temperature on MGL activity.

The biochemical properties (reaction temperature, thermal stability, reaction pH, pH stability, and inhibitor and activators) of purified MGL from the *Pseudomonas* sp. were estimated. The reaction mixtures of MGL were incubated at temperatures 20 to 60 °C, and the activity of the enzyme was calculated by standard test. The reaction temperature profile (Fig. 3), indicates that purified MGL exhibited the maximum activity at 37 °C after 30 min of incubation, with an obvious decrease in the enzyme activity at higher incubation temperatures. At an incubation temperature of 60 °C, the enzyme activity was reduced from 96.33U/ml to 56U/ml (about 41.5%) compared to the optimum reaction temperature (37 °C).

The thermal stability of the purified MGL from *Pseudomonas* sp*.* has been determined by keeping the enzyme without substrate at 4, 20, 37 and 50 °C for 30, 60, 90 and 120 min, then assessing the residual enzymatic activity. Remarkably, the catalytic efficiency and enzyme structural stability sharply decreased using the higher pre-incubation temperature. The overall thermal kinetic parameters of MGL from *Pseudomonas* sp*.* are summarized in Table 3. At 4 °C, the T1/2 of the purified MGL from *Pseudomonas* sp. was 349.3 h, ensuring the proximity of storage stability of *Pseudomonas* sp. MGL. Meanwhile, T1/2 of MGL from *Pseudomonas* sp. at 20 °C (101.2 h) showed 71% decrease in enzyme activity, while at 37 °C decreased by 88.2% with T1/2 value as (20.66 h). After incubation at 50 °C, 98.5% of MGL activity was lost with T1/2 (5.39 h). In conclusion, the thermal denaturation rate of the MGL from *Pseudomonas* sp. displayed an obvious high conformational stability towards increased storage temperatures. At 37 °C, the thermal denaturation rate (Kr) of MGL from *Pseudomonas* sp. was 0.59x10-3 min. The half-life temperature (*Tm*) of the MGL of *Pseudomonas* sp. was 101.3 °C after 60 min by about 1.18 folds increment of structural stability. Meanwhile, recorded *Tm* values were 79.54 °C after 90 min and 60.34 °C after 120 min. Meanwhile, after 120 min, the maximum thermal denaturation rate (Kr) of the tested enzyme (0.59x10-3 min & 5.16 x10-3 min) was observed at 37 °C & 50 °C, respectively.

**Table 3:** Thermal Kinetic parameters of the purified MGLs from *Pseudomonas* sp.

***Pseudomonas* sp*.***						
**T^o^C**	*T_1/2_* (h)*	*Kr* (min)**	***Tm* (°C)**			
			**30 min**	**60 min**	**90 min**	**120 Min**
4	349.3	0.00041	125.9	101.36	79.54	60.35
20	101.2	0.00044				
37	20.66	0.00059				
50	5.39	0.00516				

- Kr. *Tm =* ln (At/Ao) where Ao and At are the specific activity of MGL at zero and *t* time respectively.

*Half-life time (T1/2) was defined as time which the enzyme loses 50% of its initial activity after preheating without substrate at each temperature degree.

**Thermal denaturation rate (Kr) was known as the rate at which enzyme activity decreased logarithmically over the time at each temperature.

#### 3.3.2 Optimal reaction pH, pH stability and pH precipitation profile

**Fig. 4:**
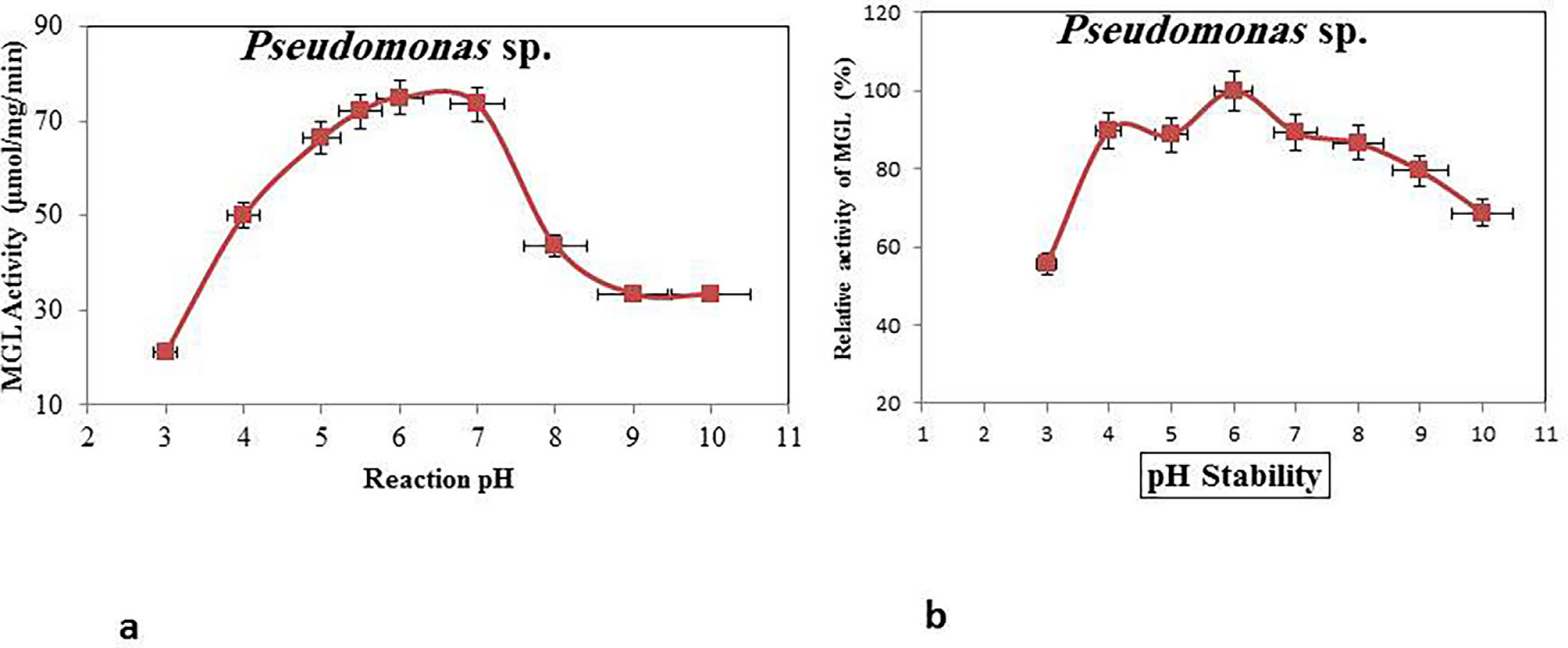
Effect of pH values on activity of purified MGL from *Pseudomonas* sp. a) Activity of MGL at different reaction pHs; b) pH stability of the purified MGL.

The purified MGL activity was assessed in response to various pH values of the reaction mixture. From the results **(**Fig. 4**a**), the highest enzyme activity was reported at pH 6.0–7.0, with a marked decrease in activity at pH 3.0 and pH 10.0. The MGL activity was reduced by about 80% at pH 3.0, suggesting the denaturation of catalytic subunits of MGL or dissociation of enzyme PLP, consequently forming inactive apo-MGL.

The purified MGL pH stability was assessed by keeping the enzyme at various pH (3–10) for 3 h at 4 °C, then detecting the MGL residual activity. The pH stability pattern (Fig. 4**b**) revealed that maximum activity was recorded at pH value 6. Meanwhile, obvious reduction of enzyme activity (45–20%) was detected at pH 3.0 and pH 9.0, assuming the drastic effect of acidic pH on the enzyme conformational structure than the alkaline one **(**Fig. 4**b).**

Purified MGL enzyme was dissolved in buffers adjusted at pH values from 3 to 10 for 24 h at 4 °C. Thereafter, the precipitated protein was estimated where maximum protein concentration was determined at pH 5 (Table 4) confirming it as the isoelectric point (*pI* = 5).

**Table 4:** Determination of *iso*-electric point (*pI*) of purified MGL from *Pseudomonas* sp*.*

**pH value**	**ppt Protein concentration mg/ml**
**3**	0.04
**4**	0.05
**5**	0.08
**6**	0.06
**7**	0.034
**8**	0.052
**9**	0.06
**10**	0.07

#### 3.3.3 Relative activity of MGL from *Pseudomonas* sp*.* towards inhibitors and amino acid analogues

In a pre-demetallizing step, the enzyme was treated with 50 mM Tris-HCl with 1.0 mM EDTA for 2 h, then amended with various cations namely Ba^2+^, Cu^2+^, Zn^2+^, Fe^3+^, Ca^2+^, Hg^2+^, Al^3+^, Na^+^, K^+^, and Mg^2+^ at 1 mM total concentration. After incubation, the enzyme activity was determined by the standard test. There was no significant effect on the activity of MGLs towards monovalent cations (Na^+,^ and K^+^), divalent cations (Ba^+2^, Ca^2+^, Mg^+2^, Zn^+2^, Hg^+2^) and trivalent cations (Al^+3^and Fe^+3^), adjusting for the demetallized MGLs residual activity (Table 5). The lack of positive effect of the tested cations on the MGL activity ensures the non-metallic identity of the purified MGLs.

**Table 5:** Relative activity of purified MGL from *Pseudomonas* sp*.* towards inhibitors and amino acid suicide analogues.

**Inhibitors (1 mM)**	**Relative MGL enzyme activity**
**Control**	**100**
**Apo-MGL**	**85**
**ZnCl_2_**	**82.5**
**CuSO_4_**	**51.9**
**CaCl_2_**	**91.7**
**FeCl_3_**	**92.5**
**HgCl_2_**	**74.9**
**NaCl**	**90.9**
**KCl**	**82.8**
**AlCl_3_**	**90.6**
**MgSO_4_**	**38.3**
**K_2_Cr_2_O_4_**	**90.4**
**BaCl_2_**	**34.6**
**H_2_O_2_**	**26.5**
**Hydroxylamine**	**32.5**
**Guanidine thiocyanate**	**22.4**
**2-Mercaptoethanol**	**12.9**
**Iodoacetate**	**60.9**
**MBTH**	**28.9**
**DTNB**	**12.7**

The enzyme activity was strongly reduced by H_2_O_2_ which might be due to its oxidizing effect on the structural amino acids and /or denaturing the molecular catalytic structure of the methioninase enzyme.

The functional domains of tested MGL were identified by the amino acid suicide analogues namely Hydroxylamine, 2-Mercaptoethanol, Guanidine thiocyanate, Iodoacetate and MBTH, DTNB (Table 5). The MGL activity was dramatically decreased by about 87% in response to 2-Mercaptoethanol and DTNB that might be because amino acids with surface thiols have been bounded to active sites of the enzyme. The residual enzyme activity of purified MGL varied from 22.4 to 32% in the case of Guanidine thiocyanate, MBTH and hydroxylamine due to the possible dissociation of pyridoxal 5-phosphate coenzyme as well as denaturation of enzyme molecular subunit structure causing the enzyme subunits to disassemble. The purified MGL retained about 60% of its initial activity in response to iodoacetate ensuring it as the least enzyme inhibitor of the tested analogues.

#### 3.3.4 Kinetics and catalytic properties of the purified MGL from P*seudomonas* sp.

The affinity of MGL for γ-elimination and deamination reactions of various amino acids namely L-cysteine, DL-homocysteine, L-tyrosine, L-asparagine, L-tryptophan, L-ornithine, L-arginine, L-phenylalanine and D-glycine was evaluated regarding L-methionine as blank. In the substrate specificity profile (Table 6), the purified MGLs displayed the maximum demethiolating and deaminating activities towards L-methionine, preceded by L-cysteine and DL-homocysteine. However, the MGL displayed a relative deaminating activity towards different amino acids by about 5–20%. The relative MGL activity from *Pseudomonas* sp*.* was approximated by 72.8 % towards L-cysteine and DL-homocysteine, as revealed by the demethiolating activity. The MGL exhibited a lower affinity for aromatic amino acids (L-tryptophan, L-arginine, L-tyrosine) with regard to methionine as standard.

**Table 6:** Substrate specificity of MGL from *Pseudomonas* sp*.*

**Substrate**	**MGL activity**	
	**Relative activity of MGL by DTNB assay (%)**	**Relative activity of MGL by Nessler’s assay (%)**
L-Methionine	100	100
L-Cysteine	85.4	84.5
DL-Homocysteine	50.9	48.9
L-Ornithine	–	31.9
L-Phenylalanine	–	26.8
L-Glycine	–	23.7
L-Asparagine	–	14.9
L-Tryptophan	–	10.3
L-Arginine	–	9.0
L-Tyrosine	–	8.6

Further kinetic properties for the purified MGL have been evaluated towards L-cysteine compared to L-methionine as the standard substrate. The kinetic and catalytic parameters; maximum velocity (*V_max_*), Michalis-Menten constant (*K_m_*) and turnover number were summarized in Table 7. The affinity of MGL of *Pseudomonas* sp*.* was 4.9 mM for L-methionine and 6.3 mM for L-cysteine. The turnover number (*K_cat_*) of MGL of *Pseudomonas* sp*.* was 53.1 s^−1^ for the L-methionine substrate and 34.9 s^−1^ for L-cysteine. The obtained kinetics parameters revealing high catalytic efficiency of MGL from *Pseudomonas* sp. for L-methionine as a substrate might be due to the molecular conformational structure and active site orientation of purified MGL enzyme.

**Table 7:** Kinetics of the purified MGL from *Pseudomonas* sp.

**substrate**	**Kinetics parameters**			
	***K_m_*_(mM)_**	***V_max_*** (μmol/mg/min)	***K_cat_* (_S_^-1^)**	***K_cat_/K_m_*** (mM^-1^s^−1^)
**L-Methionine**	**4.9**	**36.5**	**53.1**	**10.83**
**L-Cysteine**	**6.33**	**27.6**	**34.9**	**5.51**

#### 3.3.5 *In vitro* anticancer activity of the purified MGL from *Pseudomonas* sp.

**Fig. 5:**
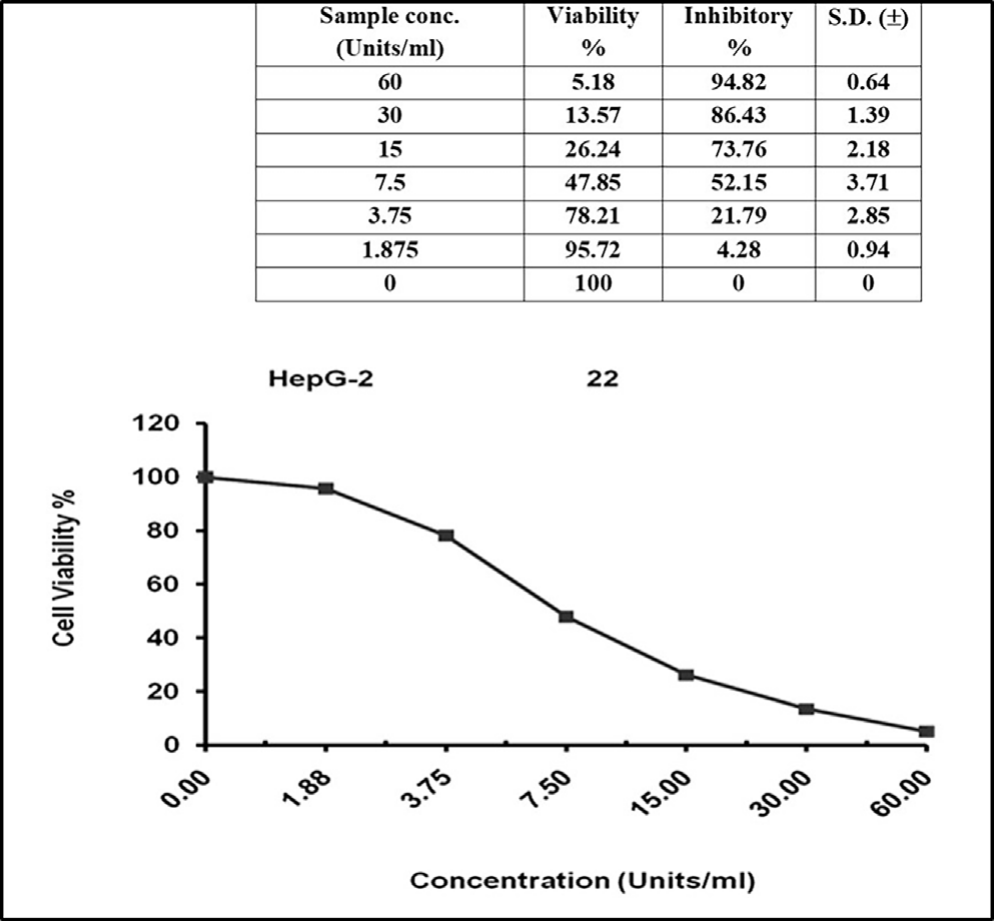
In-vitro anti-cancer activity of purified MGL of *Pseudomonas* sp. against HEPG-2 cell line.

**Fig. 6:**
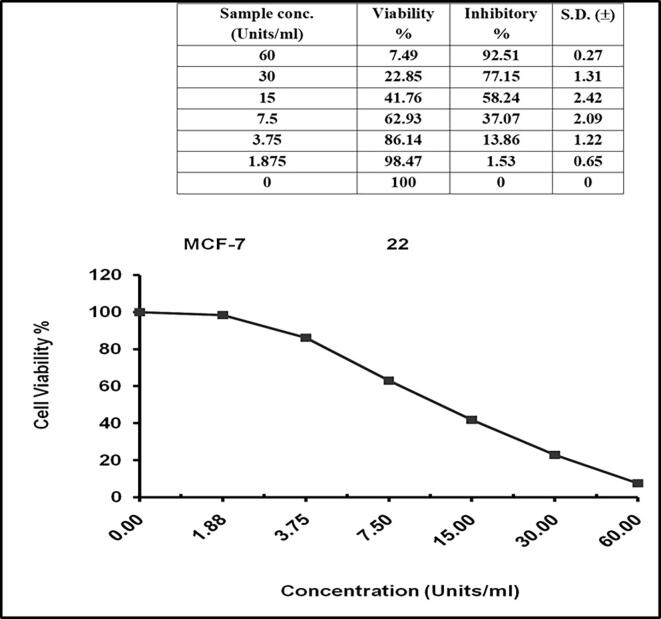
In-vitro anti-cancer activity of purified MGL of *Pseudomonas* sp. against MCF-7 cell line.

The anti-proliferative activity of the purified MGL from bacterial isolates towards breast (MCF-7) and liver (HepG-2) carcinoma cells was determined while using phosphate-buffered saline as negative control. The results in Fig. 5, indicated the IC_50_ values of MGL from *Pseudomonas* sp*.* as 7.23 μmol/mg/ min for HEPG-2 cell lines with a standard deviation ± 0.59. Meanwhile, IC_50_ values of MGL from *Pseudomonas* sp. against breast carcinoma cells (MCF-7) was 21.14 μmol/mg/min with a standard deviation **±** 0.98 **(**Fig. 6**)**.

#### 3.3.6 The *in vivo* toxicity of MGLs of *Pseudomonas* sp*.* was evaluated in mice using the biochemical characteristics of a single enzyme dose

The cytotoxic effect of the pure MGL from *Pseudomonas* sp. was determined in vivo using Swiss Albino mice. The collected Blood samples from treated mice were centrifuged and the titer of various biochemical parameters was examined in blood sera. According to the biochemical analysis (Table 8), MGL had no potent action on ALT, AST and ALP activities, with regard to controls that received injection with phosphate buffer, indicating the lack of a direct effect on the liver. Also, MGL did not show any discernible effects on the levels of albumin, globulin, the A/G ratio, total proteins, creatinine and urea during the experimented time (72 h), normalizing to controls. As revealed from in vivo biochemical analyses, the experimental animals showed no overt symptoms of MGL toxicity.

**Table 8:** Biochemical parameters *in sera* of mice in response to injection of MGL of *Pseudomonas* sp.

**Test**	**Control**	**Treated with *Pseudomonas* sp*.* MGL**	**P-value**
**ALT activity (U/L)**	64.5 ± 6.3	59.5 ± 3.55	0.196
**AST Activity (U/L)**	212 ± 32.5	234 ± 12.7	0.742
**ALP activity (U/L)**	52.5 ± 7.78	45 ± 32.5	0.069
**Albumin g/dl**	3.845 ± 0.035	3.59 ± 0.32	0.890
**Total protein g/dl**	6.37 ± 0.99	5.75 ± 0.92	0.195
**Globulin g/dl**	2.525 ± 0.063	2.16 ± 0.61	0.320
**A/G Ratio**	1.52 ± 0.028	1.71 ± 0.34	0.085
**Urea mg/dl**	37.75 ± 0.22	41.55 ± 15.6	0.905
**Creatinine (mg/dl)**	0.635 ± 0.034	0.72 ± 0.29	0.779

p-values of all examined parameter > 0.05 denoting non-significant differences.

## 4 Discussion

L-Methionine γ-lyase has received much attention for its selective activity towards various methionine-dependent cell lines (Kudou et al., 2007). The rationality of the targeted antiproliferative activity of MGL elaborates from the tumor cell's lack of activated methionine synthase, as an exceptional metabolic standard, justifying their dependence on the extrinsic plasma L-methionine (Sun et al., 2003). Methionine deprivation selectively suppresses the various biological processes such as cellular protein synthesis, and cell cycle transition (Sun et al., 2003, Kudou et al., 2007); consequently stopping tumor cells from division in the G2 phase (Tanaka et al., 1985). MGL has been extensively characterized from different bacterial and fungal sources **(**El-Sayed et al., 2012), with potential anticancer activity. However, the antigenic reactions, dissociation of PLP coenzyme from the Holo-MGL, and short biological half-life time are the main obstacles to further drug trials of this enzyme **(**Kudou et al., 2007, El-Sayed et al., 2012). Thus, screening for a potentially catalytically stable, less immunogenic MGL is the main target of this research.

The local examined bacterial isolate, identified as *Pseudomonas* sp. and submitted in Gene bank under accession number ON99389.1, is reported as a potent MGL-producing isolate. Similar studies reported the productivity of MGL from several bacterial species such as *B. linens* and *P. putida* **(**Tanaka et al., 1985, Dias, 1998**)**.

By the last stage of purification, MGL activity from Pseudomonas sp. has elevated by 1.89 folds. Similar protocols implementing the gel-filtration and ion-exchange chromatography for purification of MGL, tyrosinase, ornithine decarboxylase and asparaginase were reported (El-Sayed et al., 2017, El-Batal et al., 2021**)**. The *Pseudomonas* sp. MGL's entire molecular mass was ∼ 140–150 kD, as shown by the native-PAGE, and > 40 kD under denaturing-PAGE. So, the SDS- PAGE and native, indicated that the molecular identity of MGL has been emphasized to have four identical subunits, i.e. homotetrameric identity. The purified MGL *Pseudomonas* sp. molecular identity and subunit structure were coincident with the purified MGL from various bacterial and fungal isolates (Kudou et al., 2007, Nikodinovic-Runic et al., 2009, El-Sayed et al., 2016, El-Sayed et al., 2017). Consistently, the MGL entire molecular structure and MGL subunit structure of *A. flavipes* were ∼ 160 kilo Dalton and 49 kilo Dalton, respectively **(**El-Sayed et al., 2017**)**. Similarly, the molecular subunit structure of MGL from *Pseudomonas putida* and *B. linens* was reported to be 50 kDa under denaturing-PAGE (Kudou et al., 2007, Nikodinovic-Runic et al., 2009**)**. Coincidently, the molecular weight of bacterial MGL **(**Nakayama et al., 1988, Kudou et al., 2007**)**, protozoal and plants (Goyer et al., 2007) was between 44 and 48 kilo Dalton. The absorption spectra of the purified MGL from this bacterial isolate were scanned at 200 to 600 nm. From the absorption spectral profile, the enzyme displayed two maxima, at 280 nm (3.5) for the apo-MGL and at 420 nm (1.1) for the pyridoxal L-phosphate aldehyde's aldimine bond and the MGL lysine residues' ε- -amino group. Similarly, MGL from *A. flavipes*, *P. ovalis* and various bacterial isolates had two absorption peaks at wavelengths of 280 for the apoenzyme and 420 nm for holoenzyme, respectively (Tanaka et al., 1977, Tanaka et al., 1985, Dias, 1998, El-Sayed, 2011**)**.

Spectroscopic analyses of purified MGL enzyme produced by *Pseudomonas* sp. were determined at wavelengths 200–600 nm. Maximum absorption of UV-light at wavelength 280 nm followed by wavelength 420 nm was observed recording a high A_280/420_ ratio of 3.2. The reconstitution of the holo-MGL was monitored by the addition of pyridoxal phosphate as shown based on A_280/420_ nm ratio (Dias, 1998, Manukhov et al., 2005, Kudou et al., 2007, El-Sayed et al., 2017**)**. When PLP was added to the apo-enzyme, the ratio of A_280/420_ nm reduced from 4.01 to 1.7, revealing reassembling of active holo-MGL. The freshly purified *P. ovalis* MGL's A_280/420_ nm has consistently 3.9, then, this ratio was increased to 4.7, due to the dissociation of PLP with the repeated freezing and thawing. The apo-MGL of *P. ovalis* is unable to resume its previous activity in presence of PLP, however, the MGL of *Trichomonas vaginalis* (Lockwood and Coombs, 1991) and *A. flavipes*, in the presence of 0.1 mM PLP, >90% of its activity was recovered (Manukhov et al., 2005, Kudou et al., 2007, El-Sayed et al., 2017).

The phylogenetic analysis of peptide sequence of tested *Pseudomonas* sp. MGL showed high proximity to *Arabidopsis thaliana* AEE34271.1 and *Azorhizobium caulinodans* BAF89241.1. The peptide fingerprint of purified MGL was determined by trypsinolysis of *the* enzyme followed by LC-MS/MS. The partly peptide sequence's conserved active sites domains of *Pseudomonas* sp. MGL (Glycine- Aspartic acid-Histidine –Valine) matching with that in MGL from species of *P. asaccharolytica* AEE12456.1, *B. wiedmannii* EEK65504.1, *E. pyrifoliae* CAY73263.1, *P. gingivalis* ERJ88601.1, *B. thuringiensis* EEM45798.1, *B. cereus* EEL43444.1 and EEL04005.1.

The conserved MGL active sites are usually Tyr^196^- Gly^197^-Cys^198^, and the location of this sequence varied greatly depending on the microbial source of the enzyme, for instance, Tyr^114^-Gly^115^-Cys^116^was present in *P. putida*, Tyr^111^-Gly^112^-Cys^113^ in *Trichomonas vaginalis* and Tyr^108^-Gly^109^-Cys^110^ in *Entamoeba histolytica* (Sato et al., 2006, Sato et al., 2008). Goyer et al. (2007) reported Thr-Leu-Tyr-Gly-Cys as active site containing domains of *A. thaliana, B. linens,* and *P. putida* MGLs (preserved on other γ-family PLP-dependent C-S lyases). The MGLs from bacteria, fungi, and plants possess moieties Tyr114-Gly115 and Cys116 that substantially constitute the active site (Dias, 1998, Amarita et al., 2004, Manukhov et al., 2005, Sato et al., 2008, El-Sayed et al., 2016, El-Sayed et al., 2017**)**.

The purified MGLs from this bacterial isolate exhibited the same maximum activity at 37 °C, emphasizing the potential activity of this enzyme for in vivo therapeutic applications. Consistently, the highest activities of MGL at 37 °C were reported for the enzyme from *A. flavipes* and from other various bacterial isolates (Kudou et al., 2007, El-Sayed et al., 2016, Manukhov et al., 2005, Morozova et al., 2017, Dias, 1998**)**. Thus, the purified MGL from this bacterial isolate could have the same reactivity and conformational catalytic structure for binding with the substrate and further catalytic process. The overall thermal structural stability reported for *Pseudomonas* sp. MGL at 37 °C is consistent with MGL from *B. linens* (Dias and Weimer, 1998**)**.

The highest activity and stability pattern for the MGL from the bacterial isolate was reported at pH 6.0–7.0, with a substantial decrease in activity pH values of 3.0 and 10.0. The drastic effect at acidic pHs in range (3–6), on MGL activity might be contributed to proximity to precipitation (*pI*, 5) of the enzyme. Similar results for the pH activity and stability of MGL were reported for MGL from various bacterial (Nakayama et al., 1988, Nakayama-Hamada et al., 2005, Dias et al., 2017, Dias, 1998**)** and fungal sources (El-Sayed, 2010, El-Sayed et al., 2017). Also, the PLP's dissociation or the enzyme unfolding of tertiary structure may be the reason for the enzyme's reduced stability and inactivation at lower pHs.

The lack of positive effect of the tested cations on the MGL activity ensures the non-metallic identity of the purified MGLs. The activity of MGL from *Pseudomonas* sp*.* was dramatically reduced by 67% due to hydroxylamine which might be due to the dissociation of PLP coenzyme. A strong reduction in MGL activity was observed responsive to guanidine thiocyanate, DTNB and MBTH, ensuring the amino acid proximity of tested MGL. Coincidently, the MGL from *T. vaginalis* (Lockwood and Coombs, 1991**)**, *B. linens* (Dias and Weimer, 1998**)** and *A. flavipes)*El-Sayed, 2011**; El-Sayed *et al*., 2015;** El-Sayed et al., 2016**)** were completely blocked by carbonyl reagents; DL-propargylglycine, L-cycloserine, hydroxylamine, which are potent irreversible inhibitors to the PLP, assuring their reliance on PLP. As well as, the MGL was greatly reduced by the reaction between their sulfur amino acids and thiol reagents such as DTNB and iodoacetamide (Lockwood and Coombs, 1991**)**. *Lactococcus lactis* MGL activity was fully reduced by the carbonyl reagents; hydrazone 3-methyl-2-benzothiazolinone and hydroxylamine (Martinez-Cuesta et al., 2006**)**. An extreme reduction of demethiolating activity of *Achromobacter starkeyi* MGL by *p*-chloromercuri-benzoic acid, iodoacetate, semicarbazide and sodium arsenate was reported (López et al., 1997, Valdés-Santiago and Ruiz-Herrera, 2014). Consistently, the *A. flavipes* **(** El-Sayed, 2009**; El-Sayed *et al*., 2015** **and** El-Sayed et al., 2017) and *L. lactis* MGLs activity (Martinez-Cuesta et al., 2006**)** was greatly decreased using sulfhydryl compounds such as mercaptoethanol and iodoacetic acid.

The purified MGLs displayed the maximum demethiolating and deaminating activities towards L-methionine, followed by L-cysteine and DL-homocysteine. The relative MGL activity from *Pseudomonas* sp. was approximated at 85.4% towards L-cysteine and 50.4% DL-homocysteine, as revealed from the demethiolating activity. The catalytic efficiency of MGL of *Pseudomonas* sp. was higher by about 1.2 folds for L-methionine as substrate, revealing the affordable molecular conformational catalytic structure of MGL of the *Pseudomonas* sp. So, based on the kinetic properties, MGL of *Pseudomonas* sp*.* is highly affordable in therapeutic applications, for the greater affinity for L-methionine compared to amino acids that include sulfur. The lower affinity of MGL towards homocysteine and cysteine is a favorable criterion, due to the ability of normal cells to utilize DL-homocysteine for DNA methylation, polyamine synthesis, and glutathione synthesis in the absence of L-methionine (Kudou et al., 2007 and **El-Sayed *et al*., 2015**). However, MGL from *B. linens* (Dias and Weimer, 1998**)**, *P. ovalis* and *Clostridium sporogenes* (Tanaka et al., 1977**),** exhibited a higher affinity for DL-homocysteine compared to methionine as detected based on the *Km* values. MGL from *E. histolytica* (Sato et al., 2006, Sato et al., 2008), *A. flavipes* **(**El-Sayed, 2009**; El-Sayed *et al*., 2021)** had a 30–20% activity on DL-homocysteine comparing to L-methionine.

The antiproliferative activity of purified bacterial MGL was determined for the breast carcinoma (MCF-7) and liver carcinoma (HEPG-2) cell lines. *Pseudomonas* sp. MGL antitumor activity was higher towards the HEPG-2, recording IC50 as 7.23 ± 0.59 μmol/mg/min, than that recorded against MCF-7 cell lines (IC50 21.14 ± 0.98 μmol/mg/min). Similar results for the antitumor activity of the purified MGL produced by *A. flavipes* (El-Sayed, 2011, El-Sayed and Shindia, 2011, El-Sayed et al., 2014, El-Sayed et al., 2017) and *P. putida* **(**El-Sayed et al., 2016, Kudou et al., 2007, Dias, 1998, Nakayama et al., 1988**)**. Recorded IC50 values of *A. fumigatus* MGL against Hep-G2, and HCT116 were 243 ± 4.87 µg/ml (0.486 U/ml), and 726 ± 29.31 µg/ml (1.452 U/ml), respectively, ensuring its good catalytic properties along with significant anticancer activity at low concentrations **(**Hendy et al., 2023**).** Thus, reporting *A. fumigatus* MGL as a probable candidate to be applied in the enzymotherapy field.

The designed in vivo experiment using Swiss Albino mice aimed to examine whether the purified *Pseudomonas* sp. MGL had any harmful effects on physiological functions. It was found that the examined enzyme had no significant impact on kidneys or liver functions. Titers of albumin, globulin, A/G ratio, total proteins, creatinine, urea as well as ALT, AST and AlP along the experimented time (72 h), were not significantly altered from the normal level of control animals. Salim et al. (2020) demonstrated the high efficacy of Trichoderma MGL against cancer cell lines in vitro, and the safe tested dose levels (10 mg/kg and 20 mg/kg) in vivo conditions.

## 5 Conclusion

In conclusion, MGL enzyme extracted from a local soil inhabiting *Pseudomonas* sp. was purified to its molecular homogeneity by gel-filtration and ion-exchange chromatography. The obtaind molecular masses of denatured subunit (>40 kDa) and native enzyme (>150 kDa) confirmd its homotetrameric nature.The proteomics analysis of *Pseudomonas* sp. MGL displayed the peptide fingerprint showing the conserved active site domains (Glysine- Aspartic acid-Histidine –Valine), with a slight difference on the N and C-terminals, that might be attributed to the microbial source, or enzyme post translational modification process. The purified MGL enzyme showed high degree of stability at high temperatures up to 50 °C and wide range of pH values (pH 3–10). The enzyme also displayed the highest affinity for the substrate l-methionine than other tested substrates ensuring its promising activity in in-vivo applications in combatting L-methionine-dependent tumor. Significant in-vitro anticancer activity of *Pseudomonas* sp. MGL towards the HEPG-2 and MCF-7 cell lines, that did not exhibit any overt toxicity in the examined animals, demonstrating the absence of direct effect on either liver or kidney. As a result, the current study indicates that *Pseudomonas* sp. MGL has potential as a cancer treatment agent.

## Ethical statement

The ZU-IACUC committee of the Faculty of Medicine at Zagazig University examined and approved the animal study, which followed NIH rules, with approval number: ZU-IACUC/1/F/220/2022.

## Declaration of Competing Interest

The authors declare that they have no known competing financial interests or personal relationships that could have appeared to influence the work reported in this paper.
